# Local force titration of wood surfaces by chemical force microscopy

**DOI:** 10.1007/s10570-021-04342-3

**Published:** 2021-11-27

**Authors:** Claudia Gusenbauer, Karolina Peter, Etienne Cabane, Johannes Konnerth

**Affiliations:** 1 Institute of Wood Technology and Renewable Materials, Department of Material Sciences and Process Engineering, BOKU-University of Natural Resources and Life Sciences Vienna, Konrad-Lorenz-Straße 24, 3430 Tulln, Austria; 2 Institute for Building Materials, ETH Zürich, Stefano-Franscini-Platz 3, 8093 Zürich, Switzerland; 3 EMPA – Swiss Federal Laboratories for Materials Science and Technology, Überlandstrasse 129, 8600 Dübendorf, Switzerland

**Keywords:** Adhesion, Atomic force microscopy, Chemical force microscopy, Titration, Wood

## Abstract

Chemical force microcopy, a variation of atomic force microscopy, opened the door to visualize chemical nano-properties of various materials in their natural state. The key function of this method is given by translating adhesion forces between a functionalized tip and the sample to chemical surface behavior. In force titration, these adhesion forces are studied in different pH buffers, which allows estimating the p*K*_a_ value of the analyzed surface. Herein, we report the use of this method to study natural and chemically treated wood surfaces, which are of interest in sustainable material design. First, we show varying adhesion phenomena of OH- and COOH-functionalized tips on native spruce wood cells. Then, we demonstrate how peak force tapping with chemically functionalized tips can be used to estimate the p*K*_a_ value of gold substrates (p*K*_a_ ≈ 5.2) and different wood cell wall layers with high spatial resolution. Additionally, the swelling behavior of wood samples is analyzed in varying pH buffers. With the applied method, chemical surface properties of complex natural substrates can be analyzed.

**Graphical abstract:**

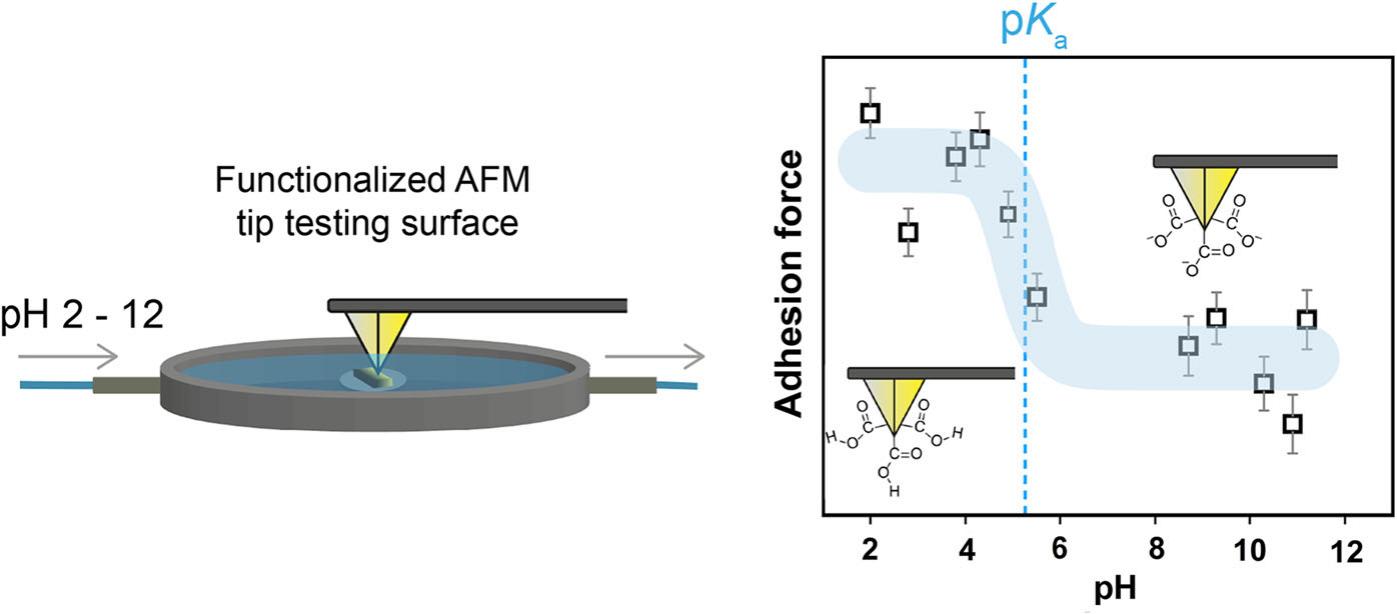

## Introduction

Products based on cellulose are of interest in sustainable material design, due to its excellent mechanical properties and high availability (Gibson 2012). Wood, as a resource for cellulose, can be used either in its natural and intact hierarchical state, or it is disintegrated into its smaller components. For all various modification steps and chemical treatments used in e.g. wood functionalization or wood disintegration for pulping, fundamental knowledge in surface chemistry is essential for the efficient preparation and integration of lignocellulose (Iglesias et al. 2020). In this regard, studies on the surface energies and interface forces that influence the surface chemistry are needed since wood responds differently to various treatments (Liu et al. 1998).

Acidic properties influence the reaction behavior of bulk wood, which can be analyzed in different ways. For instance, contact angle measurements are applied to calculate the surface free energy and for the determination of its acid and basic components (Gindl and Tschegg 2002; Gindl et al. 2004). In another method, the p*K*_a_ value of modified wood is identified by recording the pH of a solvent, in which wood flakes were stored (Balaba and Subramanian 1984). Mechanical disintegration of wood allows further possibilities to analyze the surface behavior. In such methods, it is possible to determine volatile acid contents of wood by titration of the acidic distillate of wood powder (Balaban and Uçar 2003). Additionally, potentiometric titration or conductometric titration studies the surface charges of wood pulp (Bhardwaj et al. 2004; Lloyd and Horne 1993; Katz and Beatson 1984). The challenge in these characterizations arises from the complexity of wood: the composition varies and the components are located heterogeneously in the wood scaffold (Rowell et al. 2012). The previously mentioned characterization methods are not sensitive to these local variances, which, however, are required for the optimal design of modern cellulose-based materials.

A complementary method to study surface forces with high local resolution is presented by force titration in combination with chemical force microscopy (CFM), also known as chemical force titration (Vezenov et al. 1997). This method is based on an atomic force microscope (AFM), which operates by scanning a small tip with a radius of some nanometers over the sample. In CFM, the tip is chemically modified by e.g. self-assembled monolayers (SAM) so that it terminates in well-defined functional groups (Noy et al. 1997). The movement of the cantilever that is carrying the tip is monitored and the interaction between the functionalized tip and the substrate is analyzed to image topography with chemical sensitivity (Frisbie et al. 1994). The chemical information derives from the adhesion force of the tip that performs so called force-distance measurements on the sample (Ito et al. 2010). In force titration, these adhesion forces are studied in different solution pH and thus, the p*K*_a_ value can be estimated from an abrupt change in the plotted adhesion forces (Vezenov et al. 2008; Zhang et al. 1998). Therefore, the AFM tip acts as a “nanosensor” to determine the p*K*_a_ value at the nanometric scale, which was successfully applied on SAM terminated gold substrates (Vezenov et al. 1997; van der Vegte and Hadziioannou 1997), lipid bilayers (Garcia-Manyes et al. 2006), natural hydroxyapatite (Smith et al. 2003) or poly(dimethylsiloxane) (Song et al. 2007). In lignocellulose science, pH dependent adhesion forces were acquired on softwood bleached kraft pulp (Bastidas et al. 2005). The authors of this study reported that adhesion forces between COOH-tips and cellulosic fibers decreased drastically between pH 4–6 whereas CH_3_-tips did not develop a pH dependent behavior. They also claimed that finding the same position after changing solvent was difficult, which hindered mapping locally resolved force interactions. But especially the local resolution is of importance, since adhesion forces show a heterogeneous distribution on lignocellulosic fibers (Klash et al. 2009).

In this study, we present locally resolved force titration measurements on intact wooden matrices. Initially, adhesion phenomena were analyzed with OH- and COOH-tips on native spruce wood in ambient air and aqueous surrounding. Based on these results, adhesion forces were studied in varying solution pH with COOH-tips on a model surface, i.e., COOH-functionalized gold wafer. In the developed set-up, the scanning position is kept constant and adhesion forces with COOH-tips are studied in situ with peak force tapping. In peak force tapping, the tip is tapping the sample in vertical oscillation to test the sample at high spatial resolution and high speed, which can be applied for mapping simultaneously structural and chemical properties (Alsteens et al. 2012; Gusenbauer et al. 2019). Finally, adhesion forces and topographical changes were recorded on native and carboxylated spruce wood cell walls in varying solution pH. Carboxylated wood scaffolds were chosen to increase the amount of carboxyl groups within the whole wood scaffold in expectation to see stronger contrasts in adhesion forces. Therefore, this publication demonstrates how atomic force microscopes, which are nowadays available in several research labs, can be simply adapted to acquire nanometric chemical surface information with high local resolution of heterogeneous, natural materials.

## Materials and methods

### Materials and chemicals

Sapwood of spruce wood (*Picea abies*) from Switzerland was used. For wood functionalization, succinic anhydride and acetone were purchased from Sigma-Aldrich (St. Louis, MI, USA). Pyridine (anhydrous grade) was obtained from VWR (Radnor, PA, USA). Tip functionalization required 11-mercaptoundecanoic acid (HOOC(CH_2_)_10_SH, ≥ 95%, Sigma-Aldrich), 11-mercapto-1-undecanol (OH(CH_2_)_11_SH, ≥ 97%, Sigma-Aldrich) and ethanol (HPLC grade, ≥ 99.8%, Sigma-Aldrich). Buffer preparation in the pH range from pH 2 to pH 12 required water (HPLC plus grade, Sigma Aldrich), monobasic sodium phosphate (NaH_2_PO_4_ anhydrous, ≥ 99.0%, Sigma-Aldrich), phosphoric acid (H_3_PO_4_, 85wt%, Sigma-Aldrich), sodium phosphate dibasic dihydrate (Na_2_HPO_4_*2H2O, ≥ 99.5%, Riedel-de Haën, Seelze, Germany) and trisodium phosphate dodecahydrate (Na_3_PO_4_*12H_2_O, ≥ 98%, Sigma-Aldrich).

### Functionalization of tips and wafer

Gold coated silicon nitride tips (NPG-10, Bruker, Santa Barbara, CA, USA) and small parts (~ 3 × 3 mm^2^) of a previously broken gold-coated silicon wafer (AUSW-51, Ø 2″, ~ 50 nm gold layer, NanoAndMore, Wetzlar, Germany) were cleaned for 20 min in an UV/Ozone-cleaner (UV/OZON ProCleaner, Bioforce Nanosciences, Salt Lake City, UT, USA) to remove organic contaminations. Directly after cleaning, tips and wafer were placed in a thiol solution overnight. For COOH-tips, the material was placed in a 1 mM solution of 11-mercaptoundecanoic acid in ethanol. For OH-tips, the material was placed in a 1 mM solution of 11-mercapto-1-undecanol in ethanol. After functionalization, the tips and wafers were removed from the functionalization solution and dipped in ethanol and water to remove excess functionalization chemicals. The success of the thiol functionalization was verified with contact angle measurements in preliminary tests on the thiol coated gold wafer.

### Functionalization of wood

Five wood blocks (1 × 1 × 0.5 cm^3^) were cut out of spruce wood and were chemically treated to increase the amount of carboxyl groups within the whole wood scaffold. A detailed publication can be found following a previously reported publication (Vitas et al. 2018). For this purpose, the wooden blocks were dried at 65 °C before they were immersed in the functionalization solution overnight in a flask equipped with a reflux condenser. The functionalization solution was prepared by adding succinic anhydride in 3 molar equivalent to wood glucopyranose unit (molecular weight = 162 g/mol) in 15 mL pyridine. The next day, the wood blocks were heated up in the solution at 65 °C for 2 h. Subsequently, the wood blocks were removed from the solution and submerged in acetone several times before they were dried again at 65 °C. This wood modification yielded a weight percentage gain of 15.8%, which is defined as the change in dry weight before and after the wood functionalization. The calculated degree of substitution was 0.17 per accessible OH-groups assuming a 50% cellulose content in spruce wood and that one hydroxyl group of the anhydroglucose unit reacts. The resulting material is referred to here as COOH-spruce. Wood samples which were not functionalized are referred to here as native spruce.

### Wood sample preparation

**Fig. 1:**
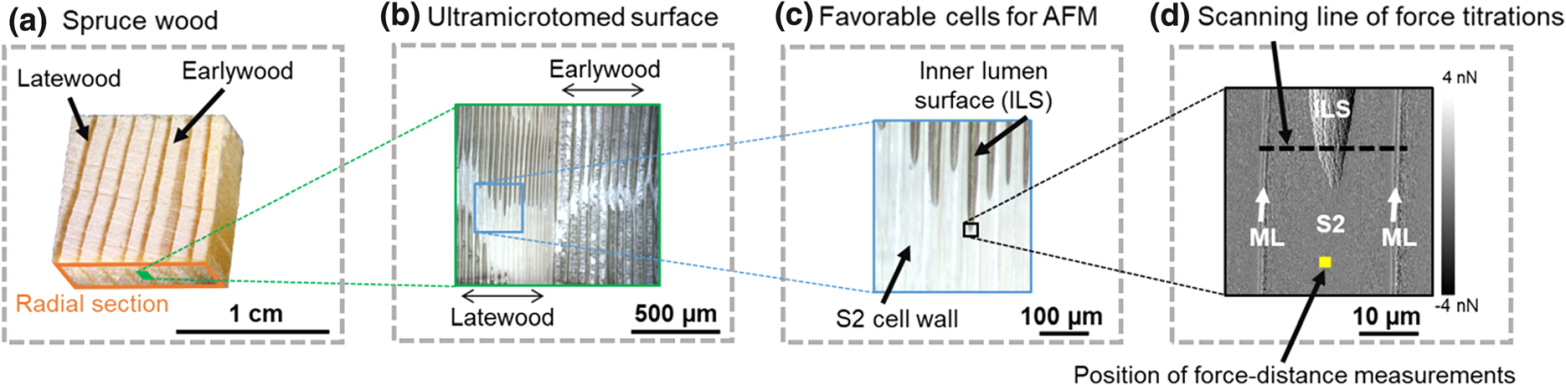
**a** Samples were prepared from both, native spruce and chemically treated spruce wood. **b** Smooth radial wood sections were ultramicrotomed and clear structural differences can be seen between earlywood and latewood. **c** The end of a cut-open cell wall/lumen in latewood shows a plane topography, which is required for AFM studies. **d** AFM error image of a wood cell wall shows the position of the force-distance and force titration measurements (S2-secondary cell wall S2, ILS-inner lumen surface, ML-middle lamella)

Polished surfaces were prepared with an ultramicrotome (Ultracut-R, Leica, Wetzlar, Germany). For this purpose, small pieces were removed from the wood blocks with a razor blade and glued onto metal disc holders. Afterwards, the wood sections were microtomed with three different knives successively, i.e., Trim 45, Histo and Ultra-AFM knife (DiATOME, Nidau, Switzerland). In this study, we polished and analyzed the radial wood surface (Fig. 1a).

### CFM—force-distance measurements.

Force-distance measurements were performed with an atomic force microscope (Dimension Icon, Nanoscope V, Bruker, Santa Barbara, CA, USA). An OH- or COOH-tip was fixed on the AFM scanner. We used cantilever A (triangular shaped, nominal spring constant = 0.35 N/m, nominal tip radius = 30 nm, resonance frequency 65 kHz) of the AFM probe for all measurements. The deflection sensitivity was calibrated on a sapphire sample and the spring constant was acquired by the built-in thermal tune method (Hutter and Bechhoefer 1993). After calibration, a freshly microtomed native wood sample was placed in the fluid chamber on the AFM sample stage and a plane wood cell wall was selected in the latewood area (Fig. 1b + c). The force-distance measurements were then performed in the center of the secondary (S2) cell wall (Fig. 1d). On a scan size of 1 µm^2^, 64 force-distance measurements were performed with 512 samples per ramp. The ramp size was 400 nm or 1 µm for measurements in water or air, respectively, with a forward velocity of 1 µm/s. Measurements in water were performed on the same area as the measurements in ambient air, after the sample was submerged 20 min in the liquid. The acquired force curves were averaged and the adhesion forces were analyzed. The adhesion force is defined as the maximum force that is needed to pull the tip away from the surface.

### CFM—force titration

**Fig. 2:**
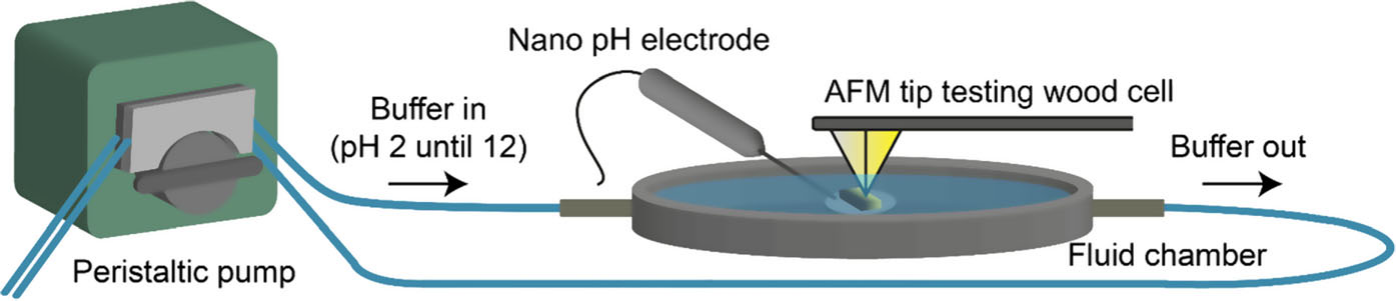
Operational scheme of force titration (not to scale). A chemically modified AFM tip acquires adhesion forces on the sample while phosphate buffers are gradually exchanged by a peristaltic pump. The pH level in the fluid chamber is recorded with a pH electrode

Force titration measurements were performed on COOH-functionalized gold wafers, native spruce and COOH-spruce specimens. In the force titration set-up, adhesion forces were acquired in buffer solutions at different pH values with an atomic force microscope (Dimension Icon, Nanoscope V). Therefore, phosphate buffers with a concentration of 0.01 mol/L were prepared ranging from pH 2 to pH 12. At first, the sample was placed in the middle of the AFM fluid chamber (Fig. 2), which was floated with a phosphate buffer of pH 2. The ambient temperature was 23 °C. Wood samples were allowed to stabilize for 20 min. Subsequently, peak force tapping scans of 4 × 512 pixels (line scans) were performed with COOH-tips mapping the cell wall from one adjacent middle lamella to the other middle lamella (Fig. 1d).

The same scanning settings were used for force titrations on COOH-functionalized gold wafers. Line scanning was done in peak force tapping mode (PeakForce Quantitative Nanoscale Mechanical Mapping, Bruker, Santa Barbara, CA, USA) with the following measurement settings: peak force frequency = 1 kHz, peak force set-point = 5nN, disabled y-scan axis, scan rate = 0.1 Hz, peak force amplitude ~ 100 nm, gain ~ 3 and lift height ~ 25 nm. After the line scan at pH 2 was finished, phosphate buffer of pH 4 was added to the AFM fluid chamber with a peristaltic pump (Ismatec Reglo ICC, Cole-Parmer, Wertheim, Germany) using chemical resistant tubes (Tygon LMT-55, Ø 1.42 mm, Cole-Parmer, Wertheim, Germany). The pH change in the fluid chamber was monitored with a pH-electrode (InLab Nano, Mettler-Toledo, Greifensee, Switzerland), which was placed next to the analyzed surface as close as possible. In this way, the pH was increased step by step by adding buffer with a higher pH value until the final pH of 12. Pumping was stopped when the targeted pH value was stable and adhesion forces were acquired. Adhesion forces of different cell wall areas, i.e., inner lumen surface (ILS) and the adjacent secondary (S2) cell wall layers, were extracted by a mask tool in the scanning probe microscopy analysis software Gwyddion (Nečas and Klapetek 2012). Finally, the average adhesion forces together with the mean absolute deviation were plotted against pH and a sigmoidal curve was fitted by a Boltzmann function in OriginPro software (coefficient of determination is displayed as R^2^). We interpreted the inflection point of the sigmoid curve as the p*K*_a_ value of the analyzed sample. Additionally, the swelling behavior from the corresponding topographical lines were analyzed. For this purpose, one single line of the cell wall topography image was extracted and plotted in OriginPro. The vertical height distance between the maximum Z value in the secondary cell wall and the minimum Z value in the adjacent middle lamella was calculated from each line scan per pH value. In this way, a maximum vertical height distance was acquired for each titration cycle which is indicated as “max. swelling” in Fig. 6b. The ratio of the vertical height distance at a certain pH and the maximum vertical height distance is defined as the swelling value and was calculated for each line scan per pH value.

## Results and discussion

### Force-distance measurements on the secondary cell wall of spruce wood

Before performing force titration measurements, force-distance curves were analyzed in preliminary tests to study adhesion phenomena on wood depending on the tip functionality. Therefore, adhesion forces were acquired with OH-tips and COOH-tips on the secondary wood cell wall as indicated in Fig. 1d by 64 simple force-distance measurements in an area of 1 × 1 µm^2^.

**Fig. 3:**
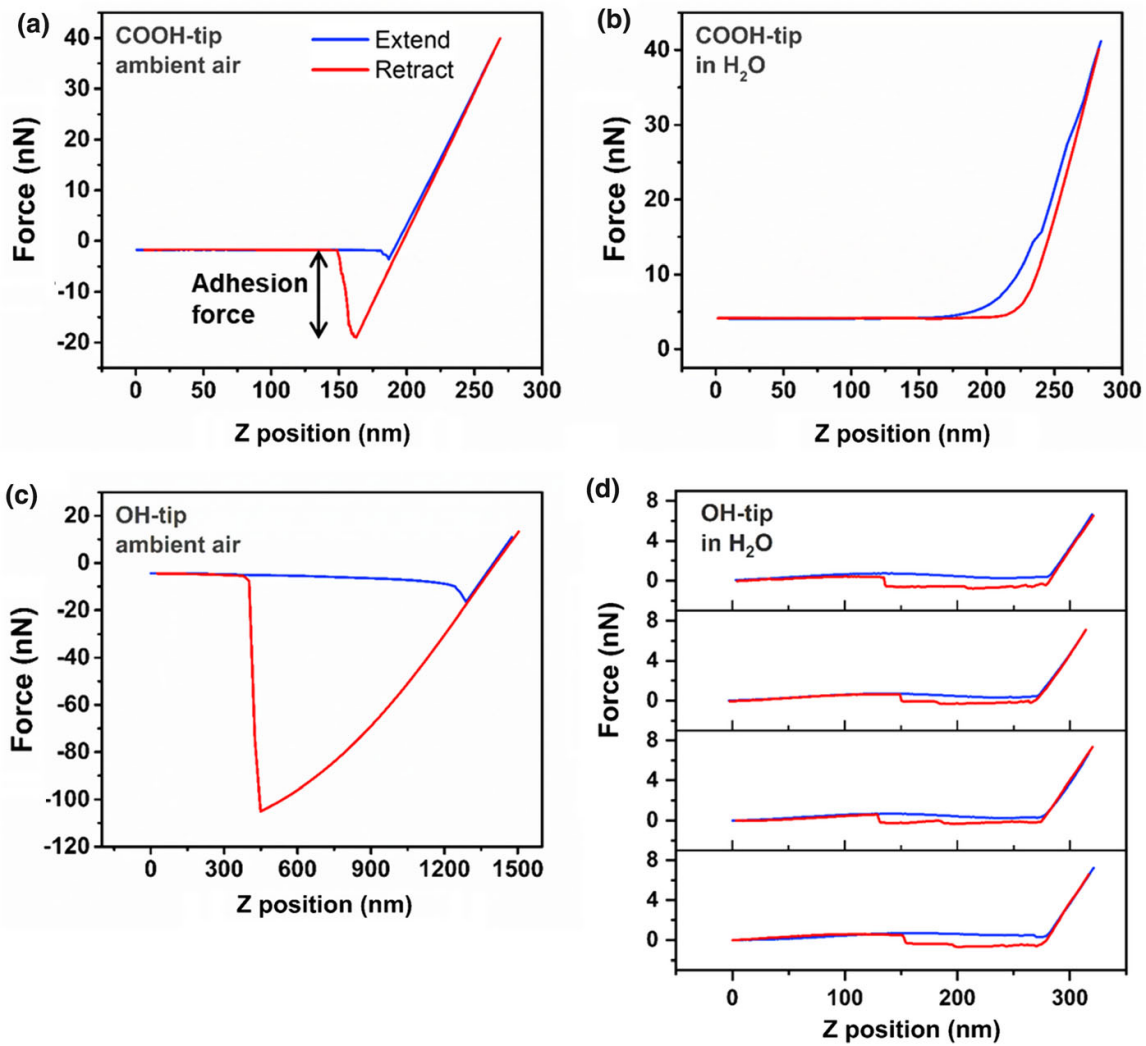
Force-distance measurements reveal adhesion phenomena occurring on native spruce wood when tested with differently functionalized tips in ambient air or water. **a-c** 64 averaged force–distance measurements. **d** Four representative force-distances curves showing rupture events in the retraction movement

For this purpose, the radial section of native spruce wood was ultramicrotomed and tested with different tips in ambient air or aqueous surrounding. Varying adhesion phenomena were occurring on wood substrates depending on the applied tip functionality and the selected surrounding (Fig. 3). We could observe that native spruce developed large adhesion forces towards OH-tips of about 100 nN in ambient air (Fig. 3c). In these force curves, a clear pull-off force can be identified in the retraction movement of the tip. Subsequently, the wood sample was submerged in water for 20 min and force-distance measurements were performed on the same position. In this configuration, the adhesion forces get drastically lower and the force profile on retraction changed behavior as indicated by the change in shape of the retraction curve. Several rupture events influenced the pull-off force (Fig. 3d). COOH-tips developed adhesion forces of approximately 17 nN towards native spruce wood in ambient air (Fig. 3a). When submerged in water, low or hardly any adhesion forces were measured with COOH-tips on the same position and there were no rupture events as seen with OH-tips (Fig. 3b).

On a freshly cut wood surface, primarily OH-groups are arising from mainly cellulose, hemicellulose and lignin polymers, which bind water molecules from the ambient atmosphere (Berthold et al. 1996). Therefore, large adhesion forces in ambient air can be explained by high capillary forces between hydrophilic tips and a layer of water molecules bound on the wood surface (Sedin and Rowlen 2000; Noy et al. 1997). The retraction movement of the tip is clearly depending on the tip functionality, when the measurements were performed in water (Fig. 3b + d). OH-tips exhibited saw-tooth like retraction behavior on native spruce wood with rupture distances of about 100–150 nm. In fibril structures, this behavior can be explained by the AFM tip that picks up molecules from the surface of the sample, which are gradually pulled away and disentangled from the substrate until they rupture from the tip or eventually from the sample (Li et al. 2016; Ahola et al. 2008). In terms of wood fibrils, the AFM tip shows multiple rupture events in the retraction due to microfibrils that bind to the tip when they come in contact and are peeled from the surface (Yan and Li 2013; Furuta and Gray Derek 1998). We assume that the microfibrils in swollen state are better accessible to the AFM tip to pick up and peel the fibrils from the surface. Unlike OH-tips, COOH-tips showed hardly any adhesion forces in water. First, capillary forces are eliminated compared to the measurements in ambient air (Weisenhorn et al. 1989; Butt et al. 2005). Second, the measurements were performed at pH 7, which leads to deprotonation and negative charge of the carboxyl groups of the tip and wood cell wall components (Bastidas et al. 2005; Vezenov et al. 1997). This behavior leads to low adhesion forces because of electrostatic repulsion between the tip and the wood surface.

Since OH-tips showed varying rupture events in the force-distance measurements and CH_3_-tips did not exhibit pH dependent adhesion forces (Bastidas et al. 2005), we performed force titration measurements applying COOH-tips.

### Force titration of model surfaces

**Fig. 4:**
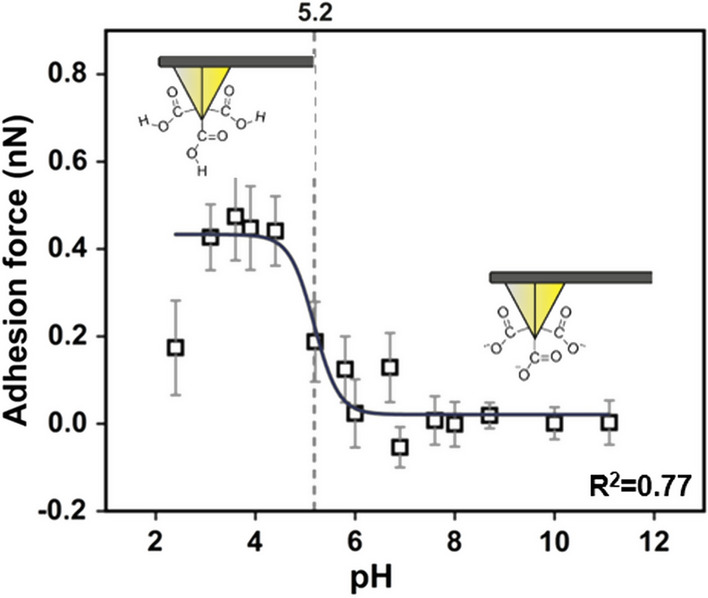
pH dependent adhesion forces between COOH-tip and COOH-functionalized gold wafer. The grey dashed line marks the position of the inflection point of the sigmoidal fit. The scheme of the tip shows schematically the surface functional groups

Adhesion forces between COOH-tips and COOH-functionalized gold wafers were studied in varying pH buffers to assure the quality of the functionalized tips and to verify the capability of peak force tapping for performing force titration measurements. A continuous increase of solvent pH was enabled by gradually adding and removing phosphate buffers with a peristaltic pump (Fig. 2). In this set-up, a functionalized AFM tip was recording adhesion forces while tapping an area of 4 × 512 pixels on the sample, referred to here as line scan. After the first line scan at pH 2 was finished, pH 4 was added to the fluid chamber and a further line scan was recorded on the same position. The adjusted pH value of the buffer was determined with a nano-electrode. In this way, line scans were acquired until approximately pH 12 while increasing the pH buffer in small steps. Average adhesion forces were extracted of each scanned area, which showed pH dependency (Fig. 4). At pH 2.4, the average adhesion force was found to be 174 pN between the COOH-tip and COOH-functionalized gold wafer. The highest adhesion force (448 pN) was measured at pH 3.9. Between pH 4 and pH 6 the adhesion force dropped and leveled at around pH 8 to 0 pN. The adhesion force data was treated with a sigmoidal fit, of which the inflection point was located at pH 5.2.

The pH dependent adhesion changes between carboxylated surfaces originate from the protonation state of the COOH groups (Vezenov et al. 1997; Vancso et al. 2005; van der Vegte and Hadziioannou 1997; Bastidas et al. 2005). Low adhesion forces at high pH can be explained by electrostatic repulsion between the negatively charged deprotonated COO^–^-tip and the deprotonated COO^–^-wafer. At low pH, the COOH groups mainly appear uncharged and adhesion forces are existing due to hydrogen bonding. Low adhesion forces around pH 2 might be caused by strong, ionic out-of-plane hydrogen bonding between neutral and ionized functional groups arising from the tip and sample, which could be prevented in the case of high electrolyte concentration buffer due to the formation of an electric double layer (Smith et al. 2000).

The location of the inflection point of the sigmoidal fit can be interpreted as an approximation of the surface p*K*_*a*_ value (Vezenov et al. 2008), as seen in Fig. 4: inflection point at pH 5.2 ≈ p*K*_a_. This finding accords with previous estimations found in research. The surface p*K*_a_ value between COOH-tips and COOH-terminated gold surfaces was previously estimated to be 5.5 (Vezenov et al. 1997) or 4.8 (van der Vegte and Hadziioannou 1997). In contrast to these publications, we observed a rather peak-shaped titration behavior than a S-shaped pattern. As described previously, this property could be caused by strong hydrogen bonding between neutral and ionized functional groups between the tip and surface.

### Force titration of native and chemically modified wood cell walls

**Fig. 5:**
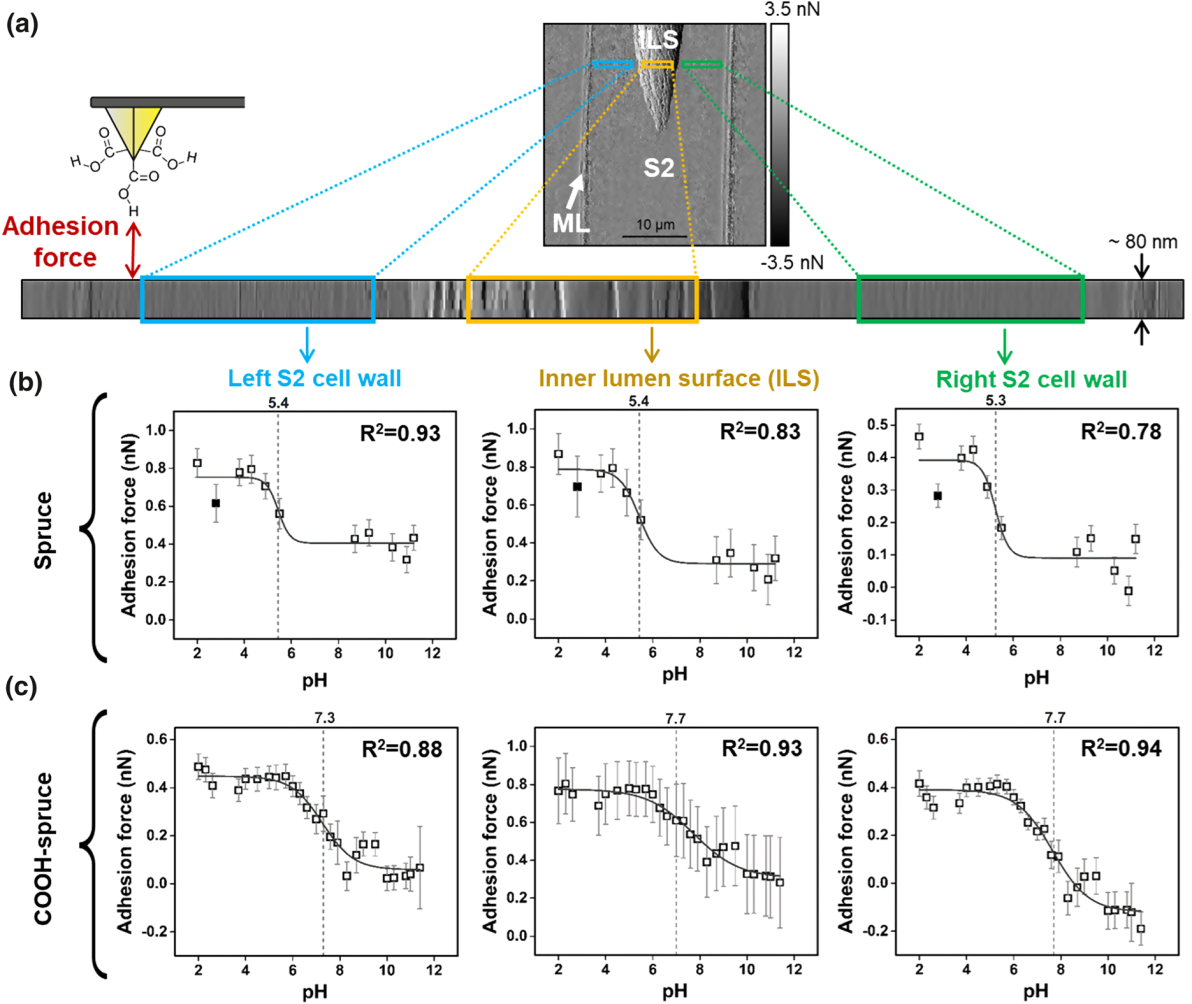
Application of the force titration principle on (un-) modified spruce wood. **a** AFM error image of a wood cell depicts the targeted scanning area (S2-secondary cell wall S2, ILS-inner lumen surface, ML-middle lamella). The colored rectangles show the area, which was used to extract and average the adhesion forces. **b** Adhesion forces between COOH-tips and native spruce show pH dependent behavior. The grey dashed lines mark the position of the inflection point of the sigmoidal fit. The filled data points correspond to the control measurement acquired 1 h after the previously performed pH series. **c** pH dependent adhesion forces between COOH-tips and COOH-spruce

After validating the capability of peak force tapping for force titration, the same experimental set-up was applied on native and chemically modified spruce wood. Line scans of approximately 0.08 × 45 µm were performed on the radial section of an ultramicrotomed cell wall area. In this way, we were able to measure a flat wood area while being able to differentiate several wood tissues, i.e., S2 secondary cell wall, inner lumen surface (ILS) and adjacent middle lamella (ML), as seen in Fig. 5a. Since the wood structure is swelling and the cell wall is expanding several micrometers, the wood samples were submerged in the first phosphate buffer (pH 2) for 20 min. Subsequently, adhesion and topography information were acquired simultaneously while continuously increasing the pH of the buffer solution. Therefore, the local distribution of adhesion was given at different pH levels. We extracted the adhesion forces at varying pH levels and differentiated between three different cell wall areas: inner lumen surface ILS, and the left and right adjacent secondary S2 cell wall layers (the positions are indicated with colored rectangles in Fig. 5a). Native and COOH-spruce wood exhibited pH dependent adhesion properties (Fig. 5b + c). It has to be noted that the two measurement series in Fig. 5b + c were obtained with two different COOH-tips. The highest average adhesion force of 868 pN was measured at pH 2 at the left S2 cell wall of native spruce wood.

**Fig. 6:**
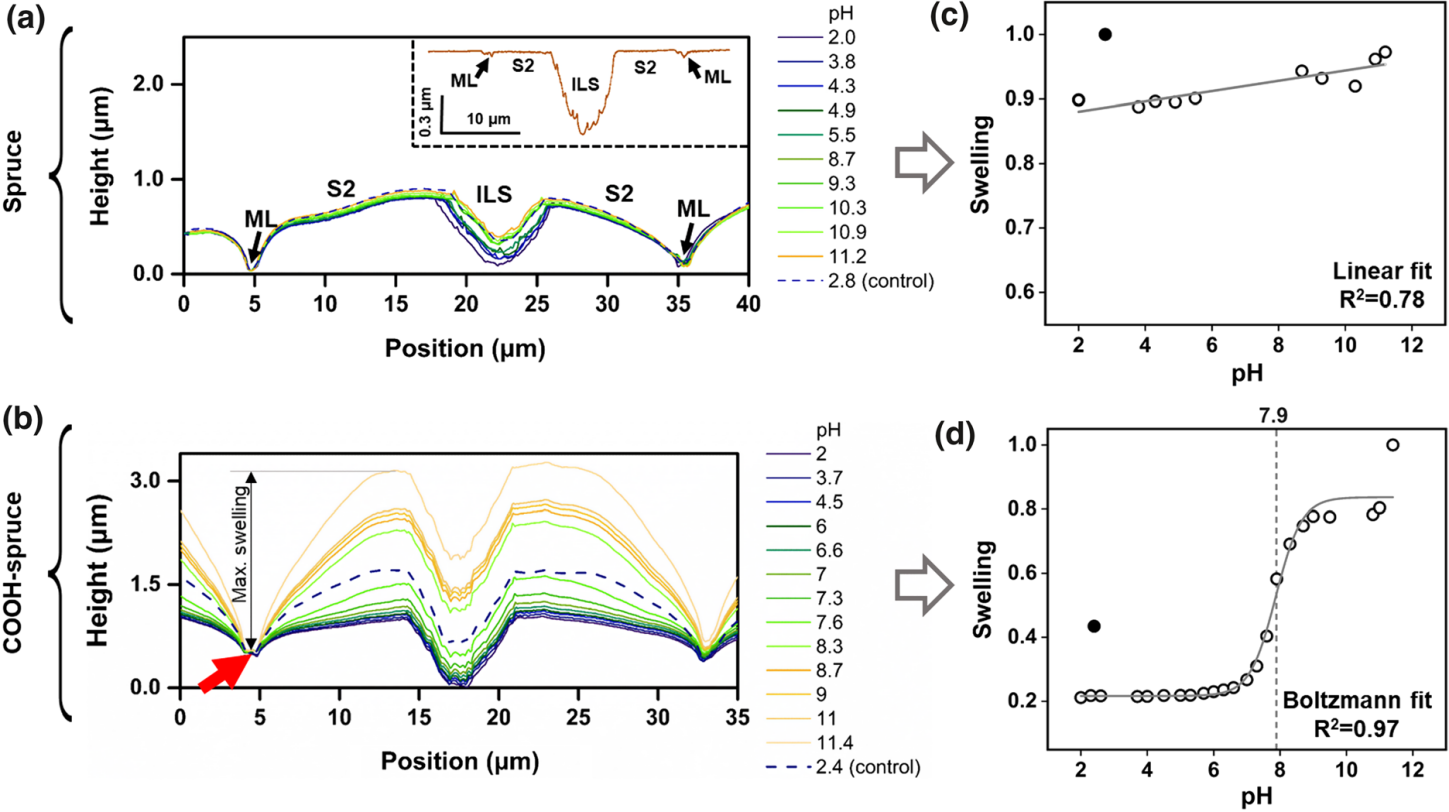
**a + b** pH dependent cross-sectional height profiles of native spruce and COOH-spruce according to the data presented in Fig. 5 (S2-S2 secondary cell wall, ILS-inner lumen surface, ML-middle lamella). The insert in **(a)** shows a cross-sectional profile of a spruce cell measured in ambient air. The red arrow in **(b)** depicts the position, on which all height profiles were aligned. **(c + d)** Swelling behavior of native and COOH-spruce. The dashed lines in **(a + b)** and the filled data points in **(c + d)** correspond to the control measurement acquired 1 h after the previously performed pH series

A drop of adhesion forces was visible starting at pH 5 and low adhesion force of about 280 pN were exhibited after pH 8. The inner lumen surface showed similar adhesion changes but the scattering of the measured forces was higher. Additionally, the right neighboring S2 cell wall showed also similar adhesion changes but the general magnitude of adhesion forces was lower on this site. Data points presented as filled squares in Fig. 5b are control values, which were recorded after the pH series was finished, to verify the reversibility of the measurements. We assume that the slightly lower adhesion values of the control measurements could originate from structural changes, which are analyzed in Fig. 6. Moreover, the long measurement duration of up to 1.5 h for one force titration cycle might harm the tip or alter the wood surface. The left and right S2 secondary cell wall of COOH-spruce showed similar trends but the general magnitude of adhesion forces was lower to native spruce. The adhesion forces measured in the inner lumen surface showed high scattering in this configuration. The data shown in Fig. 5b + c was modelled with a sigmoidal fit to estimate the p*K*_a_ value of the substrate. The position of the inflection point of the fitted line can be used to estimate the p*K*_a_ and is found to be similar in all cell wall layers of native spruce (p*K*_a_ ≈ 5.3–5.4).

The estimated p*K*_a_ was higher in COOH-spruce compared to native wood: we calculated a p*K*_a_ value of 7.7 at the inner lumen surface and the right S2 cell wall area. The left S2 cell wall area exhibited a slightly lower p*K*_a_ value of 7.3. Differences in the left and right cell wall areas could originate from the cutting direction in the “twisted” fibril alignment of the wood cell wall. The varying microfibril angle, i.e. the angle between the microfibrils and the cell axis, is between 0° and 30° in the secondary cell, also visualized by AFM (Casdorff et al. 2018), and could influence the roughness, which further influences the adhesion properties.

It has to be considered, that the magnitudes of adhesion forces are depending on the quality and dimensional properties of the functionalized AFM tip, which slightly differ from tip to tip. Therefore, comparison of the magnitude of adhesion forces from two different measurement series using two different tips needs to be done carefully, since the contact area and the amount of interaction molecules between tip and surface varies (van der Vegte and Hadziioannou 1997). Comparing Fig. 5b + c, slightly overall lower adhesion forces of COOH-spruce than native wood are visible. This effect could be caused by a slightly different tip property. Furthermore, wood materials provide an inhomogeneous fibrous structure, where surface morphology could differ from measurement position to measurement position. It was found that the wood roughness and chemical variances influence the adhesion behavior in atomic force microscopy (Jin and Kasal 2016). This is why, truly reproducible adhesion forces of wood substrates by force titration are difficult, due to the inherent heterogeneous chemical and structural differences and the sensitive force titration measurements.

The drop of adhesion forces around pH 5.4 of native spruce is according to force titration characterizations of cellulosic fibers, which exhibited a sharp change in adhesion forces between pH 4 and 6 (Bastidas et al. 2005). Considering a freshly cut wood cell wall exhibiting OH-groups to a large extend, the pH dependent adhesion behavior can be explained by attractive forces at low pH towards protonated COOH-tips and repulsive forces towards the ionized COO^–^-tips at high pH on the wood surface. The p*K*_a_ value of COOH-modified fir wood was reported to be 5.75 (Balaba and Subramanian 1984), which is lower than the p*K*_a_ of the COOH-spruce in our study. It was reported that the p*K*_a_ value of a dispersion of microfibrillated cellulose rods was depending on the ionic strength of the dispersion (the p*K*_a_ value shifted from 7.4 at 1 mM to 5.3 at 300 mM) assuming a typical p*K*_a_ value of 4.8 for carboxyl groups (Wågberg et al. 2008). In our experiments, the adhesion forces were acquired with an ionic strength of 0.01 mM, which could explain the higher observed p*K*_a_ value. Furthermore, the differences can be caused by the varying surface morphology of fir and spruce wood in combination with the differently applied methodological approaches.

To investigate topographical differences, one cross-sectional profile per pH value was extracted from the height image, which was simultaneously recorded with adhesion mapping (Fig. 6a). In both substrates, i.e. native and COOH-spruce, the position of the middle lamella was fixed, on which all pH height profiles were aligned. The samples showed different pH dependent swelling behavior in the secondary S2 cell wall area, whereas the middle lamella (ML) showed hardly any pH dependent height changes in both samples (Fig. 6a + b). The force titration measurements were performed in pH 2 to pH 12 in ascending order, and subsequently, a measurement at pH 2 (e.g. 2.8 for spruce and 2.4 for COOH-spruce) was repeated after the force titration series was finished, referred to as control measurement (dashed line in Fig. 6a + b). The insert in Fig. 6a depicts the dry ultramicrotomed cell wall structure when scanned in ambient air and shows the topographical differences between dry and swollen cell walls.

It can be seen, that native wood showed general low height expansion and the control line at pH 2.8 showed slightly higher height changes compared to the other cross-sections (Fig. 6a). Interestingly, the topographical changes were much higher in COOH-spruce compared to native spruce and the control measurement at pH 2.4 was located between the cross-sections of pH 7.3–7.6. A swelling value was defined to model the pH dependent behavior (explanation can be found in the experimental section). Native spruce expressed a linear increase in swelling (Fig. 6c), whereas COOH-spruce showed a sigmoidal behavior (Fig. 6d). Therefore, a Boltzmann fit was applied, of which the inflection point was located at pH 7.9.

The secondary wood cell wall is composed out of cellulose fibril aggregates, which are embedded in a lignin and hemicellulose matrix (Salmén 2015). The middle lamella is mainly composed out of lignin, which is “gluing” the cells together. Therefore, the high structural changes in the secondary cell wall can be explained by the liquid uptake in the fibrous wood scaffold. When the aqueous solution diffuses in the secondary S2 cell wall, H-bonding with hydrophilic wood polymers takes place leading to an expansion in the fibrillary matrix (Bossu et al. 2018; Barbetta et al. 2017). On the other hand, the middle lamella binds less water to the structure compared to cellulose fibrils due to the comparably hydrophobic and complex structural character of lignin (Rowell et al. 2012). The inner lumen surface appeared also rougher with greater height changes, which alters the contact area between tip and substrate. This condition could also explain the high scattering of adhesion forces in this area (Fig. 5b + c). Regarding pH dependent structural changes, two assumptions can be made. First, the esterification of the wood cell wall for COOH-spruce leads to decreased mechanical properties (Gusenbauer et al. 2020). Therefore, the expansion of COOH-spruce is facilitated due to the weakened wood matrix. Second, the carboxyl content in these wood scaffolds is increased and at high pH, the deprotonated COO^–^-form dominates. This ionic form leads to higher electrostatic repulsion and the carboxylated wood fibrils are able to swell more. The assumption is supported by the control measurement (dashed line in Fig. 5b), which shows that the structural expansion decreased after the solvent pH is changed from pH 11 to 4. Similar pH dependent swelling effects were observed at wood nanocellulose (Chinga-Carrasco and Syverud 2014). By introducing COOH-groups into the wood scaffold, the surface charge is changed, which is influencing the surface pK_a_ value and additionally, it was shown that an increased ionic strength leads to decreased electrostatic surface potential, which changed the p*K*_a_ value (Wågberg et al. 2008). The structural changes of about 2 µm in COOH-spruce might change the accessibility of functional groups available close to the wood surface, which could also explain a different tip-sample interaction. This combination of increased surface charge in COOH-spruce and high structural changes could explain why the estimated pKa value was higher than expected.

## Conclusion

Surface forces were studied on COOH-functionalized gold wafers, unmodified spruce and chemical treated wood (COOH-spruce). Different adhesion properties were observed depending on the tip functionality and the selected surrounding, i.e. ambient air, water or phosphate buffers ranging from pH 2–12. In force-distance measurements, adhesive interactions varied between OH-tips and COOH-tips when analyzing native spruce wood. Additionally, force titration was performed in peak force tapping mode with COOH-tips. We were able to locate pH dependent adhesion forces on COOH-functionalized gold substrates and on native and carboxylated spruce wood. In terms of wood, adhesion forces and topographical changes were extracted on the S2 secondary cell wall and inner lumen surface ILS and moreover, the surface p*K*_a_ value was estimated. Wood swelling and tip stability were identified as challenging factors. The applied method benefits from tracing the same measuring position with high accuracy and the in situ acquisition of surface properties. Peak force tapping enabled rapid data acquisition and simultaneous monitoring of structural and chemical surfaces changes. The precision of the AFM-based force titration comes along with some challenges since the method is so selective that the adhesion forces vary from position to position on heterogeneous natural materials like wood. On the one hand, the tip is very fragile and an accurate confirmation of the tip chemistry during a CFM measurement is hardly possible. On the other hand, the chemistry of wood surfaces varies naturally and these local variances may lead to changing adhesion forces. More work is required to acquire further reproducible surface forces across the wood cross-section distinguishing latewood/ earlywood or different wood cells. The developed method demonstrates to be an effective way to acquire fundamental surface information for a great variety of materials, including heterogeneous substrates such as wood.

## Data Availability

Data available on request from the corresponding author.
